# Spatiotemporal hierarchical Bayesian analysis to identify factors associated with COVID-19 in suburban areas in Colombia

**DOI:** 10.1016/j.heliyon.2024.e30182

**Published:** 2024-04-24

**Authors:** J. Cortes-Ramirez, J.D. Wilches-Vega, B. Caicedo-Velasquez, O.M. Paris-Pineda, P.D. Sly

**Affiliations:** a Centre for Data Science. Queensland University of Technology, Australia; b Faculty of Medical and Health Sciences, University of Santander, Colombia; c Children's Health and Environment Program, Child Health Research Centre, The University of Queensland, Australia; d Epidemiology Research Group, Faculty of Public Health, University of Antioquia, Colombia

**Keywords:** Covid-19, Bayesian spatiotemporal regression, Cucuta – north Santander, High-traffic roads, Mobility and connectivity, Integrated nested Laplace approximation -INLA

## Abstract

**Introduction:**

The pandemic had a profound impact on the provision of health services in Cúcuta, Colombia where the neighbourhood-level risk of Covid-19 has not been investigated. Identifying the sociodemographic and environmental risk factors of Covid-19 in large cities is key to better estimate its morbidity risk and support health strategies targeting specific suburban areas. This study aims to identify the risk factors associated with the risk of Covid-19 in Cúcuta considering inter -spatial and temporal variations of the disease in the city's neighbourhoods between 2020 and 2022.

**Methods:**

Age-adjusted rate of Covid-19 were calculated in each Cúcuta neighbourhood and each quarter between 2020 and 2022. A hierarchical spatial Bayesian model was used to estimate the risk of Covid-19 adjusting for socioenvironmental factors per neighbourhood across the study period. Two spatiotemporal specifications were compared (a nonparametric temporal trend; with and without space-time interaction). The posterior mean of the spatial and spatiotemporal effects was used to map the Covid-19 risk.

**Results:**

There were 65,949 Covid-19 cases in the study period with a varying standardized Covid-19 rate that peaked in October–December 2020 and April–June 2021. Both models identified an association of the poverty and stringency indexes, education level and PM10 with Covid-19 although the best fit model with a space-time interaction estimated a strong association with the number of high-traffic roads only. The highest risk of Covid-19 was found in neighbourhoods in west, central, and east Cúcuta.

**Conclusions:**

The number of high-traffic roads is the most important risk factor of Covid-19 infection in Cucuta. This indicator of mobility and connectivity overrules other socioenvironmental factors when Bayesian models include a space-time interaction. Bayesian spatial models are important tools to identify significant determinants of Covid-19 and identifying at-risk neighbourhoods in large cities. Further research is needed to establish causal links between these factors and Covid-19.

## Highlights

• Little is known about the risk of Covid-19 at a suburban level in large cities such as Cúcuta, Colombia.
• A Bayesian hierarchical model identified the risk factors associated with Covid-19.
• The number of high-traffic roads is the most important risk factor of Covid-19 in Cúcuta.
• This method identifies the spatial and emerging patterns of risk of Covid-19 in suburban areas.
• This methodology can be used to support health strategies and preventive policies in health.

## 1 Introduction

The Covid-19 pandemic had a profound impact in Colombia where around six million people were infected and more than 142,780 people died up to July 2023 [1]. The spread of coronavirus infection was an unprecedented challenge to the health system affecting negatively the public health infrastructure, availability of resources such as hospital space, medical staffing and intensive care units’ equipment. This implied great efforts to ensure access to diagnostics and treatments to reduce financial barriers, add beds and ventilators and manage purchases of personal protective equipment and other medical inputs [2]. Although all Colombian regions were affected, large cities with higher population were significantly impacted due to a higher demand of health services, including from surrounding areas. Cúcuta, the capital of North Santander in northeast Colombia, is the health reference centre in the region with a health infrastructure capacity that was severely impacted by high demand during most of the two first years of the pandemic [3]. The city has been historically affected by an increasing population growth, a moderate development indicator and for being the main destination of migration from neighbour Venezuela [4,5]. The incidence rate of Covid-19 in the whole of Cúcuta was 9629 cases per 100,000 people [6], however, few reports address the distribution of the disease in the suburban areas of the city.

Understanding the suburban distribution of infectious diseases in large cities is key to identify hotspots at a neighbourhood-level scale and to better identify risk factors and determinants of these infections [7]. This is important for respiratory infections where socioenvironmental conditions with uneven spatial distribution such as population density, overcrowding, mobility and connectivity and air pollution are associated with higher risk of morbidity [[8], [9], [10], [11]]. Other determinants such as poverty, unemployment, lower insurance coverage and higher income inequality have been associated with increased morbidity and mortality of Covid-19 in many regions including The United Kingdom and United States, South America and Europe [[12], [13], [14], [15], [16], [17], [18]]. However, despite the great body of research on the risk factors of Covid-19, a smaller number of studies have addressed the distribution of the disease and its determinants in suburban areas.

Previous research of Covid-19 has shown the importance of understanding its spatial patterns of spread within large cities. A higher risk of Covid-19 has been associated with suburban population proportions, greater number of health workers, and distance from larger urban settings [19,20]. Other analyses have found a higher population density to be a significant factor driving the incidence of Covid-19 morbidity and mortality [21,22]. In contrast, most studies in Colombia provide evidence about socioeconomic inequalities in COVID-19 morbidity and mortality for larger areas such as municipalities, highlighting differences in terms of age groups, sex, ethnicity and socioeconomic status [17,23,24] while some studies have analysed the association of particulate matter exposure and altitude with COVID-19 mortality [25,26]. In Cúcuta, most analyses correspond to statistics and health reports for the whole city although the counts of cases and mortality can be obtained at the neighbourhood level.

A well-established method to estimate the morbidity risk of infectious diseases in small areas such as the Cúcuta neighbourhoods, is the use of spatial statistics that incorporate the spatial distribution of the neighbourhoods to identify spatial patterns of risk while accounting for potential risk factors. Spatial analyses of Covid-19 have shown that this approach helps to detect the heterogeneity and connectivity of the population to understand its spatial patterns of risk more accurately [[27], [28], [29]]. These analyses can be expanded to incorporate a temporal component to consider inter period variations in the Covid-19 spread and the risk factors to identify spatiotemporal trends across suburban areas. A key advantage of spatial statistical analyses is the opportunity to identify specific areas and communities in need of targeted interventions to support the implementation of public health strategies and better management of health resources. This is critical in settings with high socioeconomic pressures including large cities in middle income countries, such as Cúcuta. This study aims to identify the risk factors associated with Covid-19 morbidity in Cúcuta, and map the spatiotemporal trends of Covid-19 risk between 2020 and 2022, by implementing a hierarchical Bayesian multiple regression analysis. The following sections are organised as follows: Section 2 describes the data and methods used, Section 3 presents the results of two spatial Bayesian models compared and risk maps of Covid-19, and Section 4 discuss the results and the limitations of the study, to provide some conclusions and recommendations in Section 5.

## 2 Materials and methods

The study uses the city neighbourhoods as the spatial units to implement a spatiotemporal analysis of the association of Covid-19 rate with sociodemographic factors in 8 periods corresponding to the quarters between 0101 and 04–2020 and 31-03-2022. This study was approved by the University of Santander bioethics committee (Reference: VII-FT-025-UDES, 07-02-2022).

### 2.1 Data

Data of confirmed Covid-19 cases were obtained from the municipal health department after ethics approval. These data include the date of diagnosis, age, sex and residency (neighbourhood). Each record was geocoded to a geographical area consistent with a map of the Cúcuta's neighbourhoods. The map was designed in ArcMap (v. 10.3) by matching the city's neighbourhoods according to the municipal land use plan [30] with blocks established by the National Department of Statistics —DANE, in the National Geostatistical Framework [31]. The resulting map included 245 areas of which 209 were neighbourhoods and 36 were unpopulated areas for sections of the airport, the Pamplonita river, parks and undeveloped inhabited areas (Fig. 2). Since the setting included all Cúcuta's neighbourhoods, rather than sampling, the study used all cases of Covid-19 across the first two years of the pandemic. The prevalence rate of Covid-19 for each neighbourhood was calculated as the total cases divided by the total population per period. To adjust for differences in sex and the age structure of the population, the indirect standardised rate (age-adjusted) of Covid-19 was calculated with the R package Epitools [32], for each quarter and each and both sexes as:

Covid−19_sr=OjEj

where $O_{j}=\sum_{i\;}\left({pop}_{ij}/\sum_{i}{pop}_{ij}\right)\;\left(n_{ij}/{pop}_{ij}\right)$ is the observed Covid-19 cases in the j neighbourhood. $E_{j}=\sum_{i}\;\left({pop}_{ij}/\sum_{i}{pop}_{ij}\right)\;\left(n_{is}/{pop}_{is}\right)$ is the expected count of Covid-19 cases where ${pop}_{ij}$ is the population in the i age class of the j neighbourhood, $n_{is}$ is the count of Covid-19 cases in the i age class of the standard count (the whole of Cúcuta), and ${pop}_{is}$ is the population in the i age class of standard population (the whole of Cúcuta).

Sociodemographic data included percentiles of the Index of Multidimensional Poverty (IMP) calculated as the average of the of IMP in all blocks in a neighbourhood, and four indicators of education level: the proportion of analphabetism and the proportion of people with primary, secondary and tertiary education that were calculated per neighbourhood. In addition, the crowding index was calculated as the proportion of households with 5 or more people in all blocks in a neighbourhood. The IMP and crowding index, and the education data at the block level are available only for the year 2018, with no inter quarter data, therefore there were no differences in these indicators between the eight periods.

The stringency index (percentile) was used as an indicator of the government response to control the impact of the pandemic, for which a higher value indicates stronger and tighter containment and closure restrictions. These restrictions were equally implemented across the city therefore all neighbourhoods had a similar stringency index in each quarter. The stringency index data were obtained from the Oxford COVID-19 Government Response Tracker [33]. The count and the extent of high-traffic roads were used as an indicators of urban mobility and connectivity, following the approach of Bansal et al. [34]. Data on the number and length (in mts) of secondary and tertiary highways in Cúcuta were obtained from Open Street Maps. These are the streets that traverse neighbourhoods and their main access and represent high-traffic roads [35]. Although data on high-traffic roads extent for each neighbourhood do not include measures per quarter, there were minimal or no variations in the infrastructure of these roads during the pandemic, therefore the same value was used for each neighbourhood in all periods.

To adjust for air pollution level, the average PM_10_ concentration was calculated in each neighbourhood following the method described by Cortes-Ramirez et al. [11]. In brief, an inverse distance weighting interpolation model of air monitoring stations data was implemented in ArcMap (v.10.6) to estimate average PM_10_ percentiles.

### 2.2 Statistical analysis

A Bayesian hierarchical regression model was used to estimate the morbidity risk of Covid-19 in Cúcuta after adjusting for sociodemographic factors and taking into consideration the spatial distribution of the city neighbourhoods and variations across the eight periods in 2020–2022. As multiple neighbourhoods had no Covid-19 cases in one or more periods, the distribution of the Covid-19_sr was analysed to identify whether a zero inflated Poisson distribution suited the data [36]. An excess of zeros in all periods was identified using the zero-test in R [37], therefore a zero inflated Poisson (ZIP) model was fitted with the mixture distribution described as

$$\mathrm{Pr}\left(Y=0\right)=\pi +\left(1-\pi \right)e^{-\lambda_{it}}$$

$$\mathrm{Pr}\left(Y=y_{it}\right)=\left(1-\pi \right)\frac{\lambda^{y_{it}}\;e^{-\lambda_{it}}}{y_{it}!}$$

where π is the probability of extra zeros, $y_{it}$ is the standardised count of cases, and $\lambda_{it}$ is the population for the $i^{th}$ neighbourhood in the $t^{th}$ period, respectively. The linear predictor was defined on the logarithmic scale

$$\eta_{it}=\log\left(y_{it}\right)=\alpha +\lambda_{it}{+\beta }_{x}X_{xi}+s_{i}+u_{i}+T_{t}$$

where α is the intercept, X represents the vector of sociodemographic covariates with their respective regression coefficients $\beta_{x}$ , the parameters s and u represent the spatially structured and unstructured components according to the Besag-York-Mollie (BYM) specification [38], and T is the effect of the temporal trend considering each quarter t. The spatial structure was defined as an adjacency matrix with a queen specification (i.e., including all neighbourhood sharing a border), an optimal specification as tested in previous spatial analyses of Cúcuta geographical areas [39]. To assess the effect of the spatial and temporal specifications the following models were compared:

1. a model with a dynamic nonparametric formulation of the linear predictor described as

(1) $$\eta_{it}=\log\left(y_{it}\right)=\alpha +\lambda_{it}+\beta_{x}X_{xi}+s_{i}+u_{i}+{\gamma_{t}+\phi }_{t}$$

where $\gamma_{t}$ is the temporally structured effect modelled dynamically using a random walk of order 2 defined as γ *t* | γ *t*−1*,* γ *t*−2 ∼ Normal(2 γ *t*−1 + γ*t*−2, *σ*2), and $\phi_{t}$ is the unstructured temporal effect specified with a Gaussian exchangeable prior $\phi_{t}∼\text{Normal}(0,1/\tau \phi )$ [40].

2. a nonparametric model with an interaction between the unstructured spatial and the unstructured temporal effects ($\delta_{it}$ = $u_{i}*\phi_{t}$), as introduced by Knorr-Held [40] with the linear predictor described as

(2) $$\eta_{it}=\log\left(y_{it}\right)=\alpha +\lambda_{it}+\beta_{x}X_{xi}+s_{i}+u_{i}+{\gamma_{t}+\phi }_{t}+u_{i}*\phi_{t}$$

A preliminary analysis was implemented to assess the effect of the prior on the log of the structured and unstructured spatial precisions $s_{i}$ and $u_{i}$. Four non-informative priors previously assessed in analyses of Cúcuta geographical areas [39] were used (details in the Appendix). The best-fit model for each prior was identified using the Watanabe–Akaike information criterion (WAIC), i.e., a lower WAIC value indicates a better fit [41]. Strong statistical associations were identified when the 95 % credible intervals (CI) did not cross the null value of 1.

All covariates in the models were scaled to facilitate the comparison and interpretation of the posterior mean (i.e., regression coefficients). The neighbourhood-specific posterior mean was used to map the morbidity risk of Covid-19 as once exponentiated it can be interpreted as the residual relative risk in each neighbourhood compared to the whole of Cúcuta. All models were fit in R with the R-INLA package that uses the integrated nested Laplace approximation (INLA) [42]. This is a robust alternative to computationally and time intensive simulations with Markov Chain Monte Carlo methods to produce regression estimates in analyses of spatially auto correlated data [43]. All maps were drawn with the R-package T-map [44]. ArcGis Pro (v.3.1) was used to draw 3D maps (spatiotemporal trend of risk) and to calculate and map emerging trends using the Mann Kendall trend test [45].

## 3 Results

**Fig. 1:**
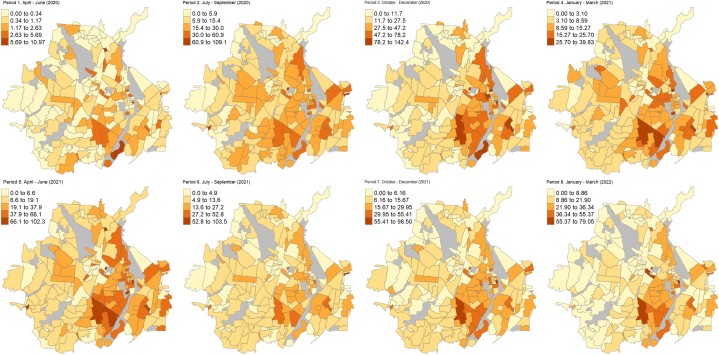
Spatial distribution of the Covid-19 standardized rate in each of the 8 periods. Grey zones represent inhabited areas.

There were 65,949 cases of Covid-19 in Cúcuta during the study period, of which 35,138 (53 %) and 30,811 (47 %) were females and males, respectively. The cases of people in ages 0–14 years; 15–24 years; 25–44 yeas; 45–64 years and 65+ years represented 5.2 %; 12.4 %; 43.9 %; 26.9 % and 11.6 % respectively. There were important variations between periods in the average of the Covid-19_sr, the Covid-19_sr in females and males and the stringency index (Fig. 1). Only small inter period differences were identified in the population density and no inter period variations in the IMP and the extent of high-traffic roads (Table 1, Fig. 1).

**Table 1:** Summary statistics of the Covid-19 rate and predictor variables in the Cucuta neighbourhoods across the study period.

	mean	sd	median	min	max	skew	kurtosis	se
Standardised Covid-19 rate	12.482	11.875	8.553	0.000	63.180	1.542	2.637	0.759
Standardised Covid-19 rate - females	12.543	11.798	8.804	0.000	66.127	1.570	3.051	0.754
Standardised Covid-19 rate - males	12.429	12.299	8.603	0.000	61.328	1.634	2.849	0.786
Multidimensional Poverty Index	21.716	15.117	20.525	0.435	68.557	0.781	0.123	0.966
Overcrowding ∗	0.051	0.026	0.062	0.000	0.087	−1.000	−0.275	0.002
Stringency Index	65.417	0.000	65.417	65.417	65.417	–	–	0.000
Analphabetism ∗	0.025	0.020	0.022	0.000	0.128	1.106	2.907	0.001
Primary education ∗	0.203	0.118	0.225	0.000	0.391	−0.389	−1.071	0.008
Secondary education ∗	0.329	0.151	0.399	0.000	0.485	−1.354	0.464	0.010
Tertiary education ∗	0.173	0.135	0.126	0.000	0.509	0.464	−1.094	0.009
PM_10_	40.598	3.296	41.135	33.363	44.907	−0.516	−0.915	0.211
High-traffic roads density∗∗	522.446	860.395	177.260	0.000	5380.513	2.600	8.003	54.969
High-traffic roads count	2.788	3.906	1.000	0.000	21.000	1.969	3.711	0.250

sd: standard deviation.

se: standard error.

∗ proportion.

∗∗ (mts per neighbourhood). Rates are expressed per 1000 people.

The spatial trend of the Covid-19_sr is shown in Fig. 1. There were important variations in the Covid-19_sr distribution between neighbourhoods across the eight periods, especially in periods 3 to 5 (Oct–Dec 2020 and Apr–June 2021, respectively) which had the highest rates. Neighbourhoods in central, central-eastern and eastern areas had higher Covid-19_sr in periods 2–5 and 7–8, while the distribution of the Covid-19_sr was more uniform in period 6.

**Fig. 2:**
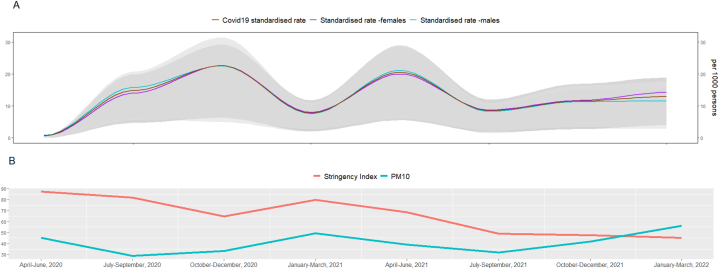
A. Smoothed trend of the Covid-19 rate for all cases and females and males across the study period. B. Temporal trend of the Stringency Index and the average Pm_10_ levels.

Fig. 2A shows the trend for the average Covid-19_sr. There was a steady increased of the Covid-19_sr until the third period to peak again in period 5 with a slight increase from period 6 to 8. This trend was closely followed by the Covid-19_sr in females and males although there were important differences in the first, third and fifth quarters and in the last period.

The trend of the stringency index and the average PM_10_ levels are shown in Fig. 2B. The stringency index had a negative trend with only one peak in period 4. There were important variations in the PM_10_ levels, with the lowest values in periods two and six, a peak in period four and an increased trend from period 6 until the end of the study period.

**Fig. 3:**
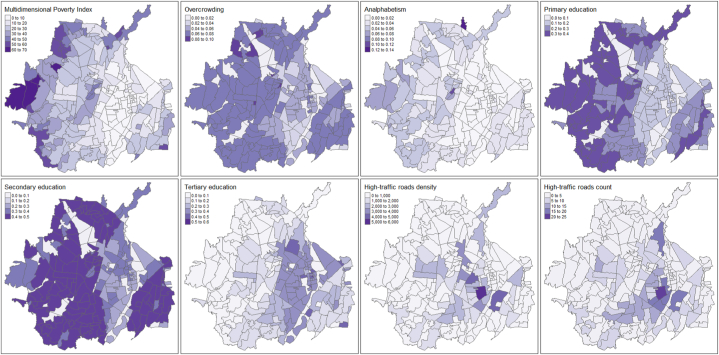
Spatial distribution of the average multidimensional poverty index (IMP), the proportion of overcrowding; analphabetism, and primary, secondary and tertiary education, and the number and extent of high roads across the eight periods.

There were no inter quarter variations of the IMP, the proportion of overcrowding, the number and extent of high-roads and the proportion of analphabetism, primary, secondary and tertiary education, since these were obtained from the 2018 census data. However, there were important differences between neighbourhoods that are shown in Fig. 3. Multiple neighbourhoods in western Cúcuta have the highest IMP as well as some neighbourhoods in northern and south-eastern areas. The most overcrowded neighbourhoods were found in north areas and some in central Cúcuta. Only two neighbourhoods in north and central Cúcuta had more than 12 % of analphabetism while the neighbourhoods with higher levels of tertiary education were in central and some western areas. There was a more even distribution of primary and secondary education with higher levels in west, north and southeast areas. Most high-traffic roads were found in neighbourhoods in central and south-eastern areas, and some neighbourhoods in northern areas of the city.

### 3.1 Bayesian spatiotemporal regression models

Table 2 shows the exponentiated fixed effects estimated in the regression models. Both models found a positive association of the high-traffic roads count and the standardised rate of Covid-19 in both females and males with the Covid-19_sr, while only model 1 identified a positive association with the IMP, the proportion of tertiary education and the average PM_10_ level, and a negative association with the stringency index. The incorporation of a space-time interaction in model 2 produced a much better fit to the data (lower WAIC) compared to model 1. The positive association of the high-traffic roads count with the Covid-19_sr identified model 2 was comparatively smaller than model 1.

**Table 2:** Exponentiated posterior mean and 95 % CI estimated in the models compared.

	Model 1	Model 2
Standardised Covid-19 rate - females	**1.181 (1.181–1.182)**	**1.31 (1.264–1.358)**
Standardised Covid-19 rate - males	**1.132 (1.132–1.133)**	**1.247 (1.202–1.294)**
Multidimensional Poverty Index	**1.293 (1.252–1.336)**	1.018 (0.896–1.156)
Overcrowding	1.044 (0.872–1.251)	1.053 (0.902–1.23)
Stringency Index	**0.49 (0.478–0.503)**	0.854 (0.556–1.31)
Analphabetism	0.973 (0.866–1.093)	1.012 (0.912–1.123)
Primary education	0.95 (0.646–1.398)	0.99 (0.715–1.372)
Secondary education	1.313 (0.967–1.784)	1.238 (0.952–1.609)
Tertiary education	**1.834 (1.248–2.7)**	1.392 (0.993–1.954)
PM_10_	**1.104 (1.102–1.106)**	0.988 (0.887–1.102)
High-traffic roads density	0.926 (0.823–1.042)	0.9997 (0.94–1.063)
High-traffic roads count	**1.2001 (1.062–1.357)**	**1.077 (1.013–1.145)**
WAIC	710169.7	21562.75

WAIC: Watanabe–Akaike information criterion.

**Fig. 4:**
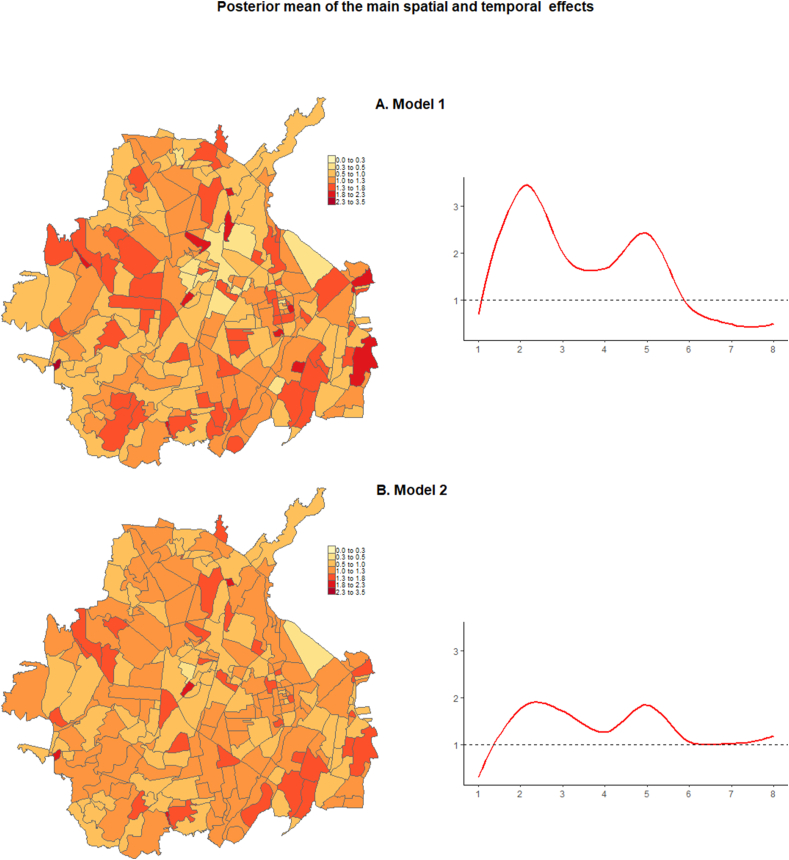
Exponentiated neighbourhood specific posterior mean of the main spatial effect (maps in the left) and exponential posterior mean of the main temporal effect (trends in the right) on the risk of Covid-19 in Cúcuta.

The maps in Fig. 4A and B show the specific posterior mean (i.e., residual relative risk in each neighbourhood compared to the whole of Cúcuta) of the main spatial and temporal effects in both models. Considering the main spatial effect, none of the models show a clustering pattern. Model 1 identifies the highest risk in multiple neighbourhoods in western, central and eastern areas while in Model 2 the highest risk is identified in three neighbourhoods in west, central, and east Cúcuta, respectively. The posterior mean of the main temporal effect in both models is shown in the trend lines in Fig. 4A and B. Both models identify a peak in the second period followed by a decrease in period 4 and another peak in period 5. In model 1, this is followed by a consistent decrease until almost the end of the study period whereas in model 2 the risk stabilises in period 6 with a slight increase from period 7 until the end of the study period, following a pattern closer to the trend of the Covid-19_sr.

**Fig. 5:**
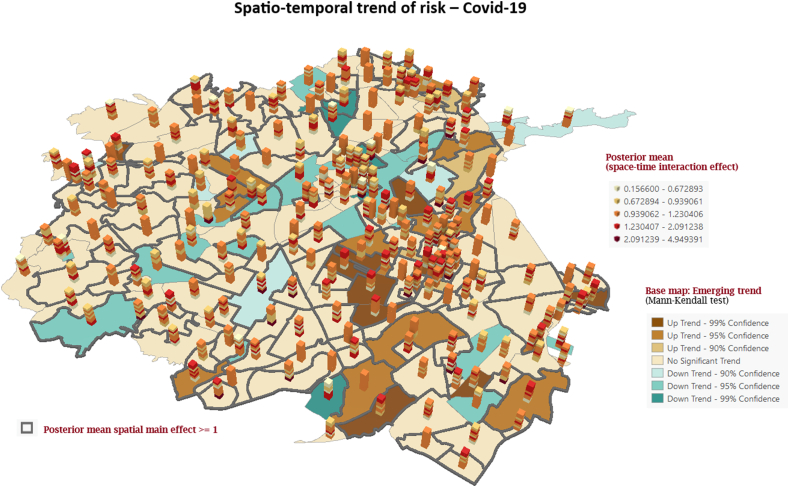
Exponentiated suburb-specific posterior mean of the space-time interaction effect and emerging trend of risk of Covid-19 in Cúcuta, from 2020 to 2022.

The incorporation of a space-time interaction allows to map the spatial distribution of relative risk of Covid-19 in each period. The neighbourhood-specific risk of Covid-19 relative to the whole of Cucuta and the overall trend estimated in model 2 is shown in in Fig. 5 (3D cylinders). There were important variations in the Covid-19 risk between periods in each neighbourhood. Whereas most neighbourhoods had an overall positive risk (posterior mean effect >1), there were important differences in the trend of risk across the eight periods. The emerging trend of risk identified in the base map in Fig. 5 shows several neighbourhoods in central Cúcuta and two neighbourhoods in south and southeast and a neighbourhood in east Cúcuta, respectively, to have the highest positive trend (increasing risk) while four neighbourhoods (two in central Cúcuta and two in the west, respectively) had the lowest trend (decreasing risk).

## 4 Discussion

This study identified the number of high-traffic roads, used as an indicator of urban mobility and connectivity, as the most important risk factor determinant of Covid-19 infection in Cúcuta, once the spatial and temporal distribution of cases and other socioenvironmental factors are taking into account. While other factors usually associated with a higher risk of Covid-19 such as poverty, education and air pollution as well as social restrictions were identified in one of the models compared, a more complex model with a spatiotemporal interaction term showed that these factors have lower statistical credibility compared with the number of high-traffic roads per neighbourhood. These outcomes highlight how spatial hierarchical models can be a useful and powerful tool to produce robust estimates of risk of Covid-19 and to identify risk factors and specific areas at risk for infectious diseases such as Covid-19 where the geographical distribution of the population is especially relevant in the disease transmission. In addition to identifying significant risk factors, the spatiotemporal regression implemented in this study allowed estimating overall and neighbourhood-specific spatial, temporal and spatiotemporal trends of risk which once mapped can be used to support decision-making in health. This is the first study to estimate and map the area-specific risk of Covid-19 in a large urban setting in the Colombian northwest.

Our findings of an increased risk of Covid-19 in areas with higher mobility and connectivity have been previously established in multiple studies in other countries [46,47]. However, measures of mobility and connectivity such as roads networks in urban settings are more precise when compared to country-level analyses, and therefore important to understand area-specific disease spread patterns [48]. We found neighbourhoods with more high-traffic roads to have an increased risk of Covid-19, something that has been identified in studies in urban settings in the United States [49], China [50], Korea [51], Australia [52] and India [28]. The findings from these studies establish that a higher mobility and influx of people can explain the higher risk of cases grow from adjacent areas. Nevertheless, our analysis found the number of high-traffic roads per neighbourhood to be a better determinant of the infection compared to other sociodemographic and environmental factors. The smaller impact of socioenvironmental factors in the Covid-19 transmission in this study can be due to the implementation of a spatial hierarchical model with a space-time interaction term. A space-time interaction considers differences in the temporal trend of the disease for each area which can be missed in a less complex model specification [43].

The results of this study suggest that if the interplay of multiple factors determinant of Covid-19 considers an interaction of their overall spatial and temporal trend in each neighbourhood, the effect of a higher number of high-traffic roads overrules the effect of higher levels of poverty and PM_10_ and stricter social restrictions on the risk of Covid-19 infection in Cucuta. Although most analyses identify a strong association of socioenvironmental factors with Covid-19 [53], previous research that found that road networks can be better moderators of the Covid-19 infection than sociodemographic factors support our findings [54]. These findings can be reached only once the distribution of the Covid-19 rate and the predictors are used in a statistical analysis than considers the spatial structure of the Cúcuta neighbourhoods which is characteristic in Bayesian spatial hierarchal models. Whereas this methodology has been increasingly used in epidemiological research, there are still many studies of Covid-19 that do not account for the spatiotemporal distribution of risk which can impact on the robustness to assessing prediction uncertainties [55]. The flexibility of Bayesian spatial models allows not only the expansion of a spatial specification to include temporal random effects to implement fully spatiotemporal models but also to assess different space-time interaction specifications [43]. We used the advantages of these models to compare two spatiotemporal specifications to establish the importance of the high-traffic road count for the risk of Covid-19 in Cúcuta.

Another outcome of our study is the mapping of risk that can be obtained from the posterior distribution of both the main spatial, and space-time interaction effects. This expands the value of this analysis from identifying the main determinants of the disease to estimate statistically what areas are disproportionally affected and the dynamics of transmission throughout the first two years of the pandemic. The local scale of the spatial areas and the long term used in our analysis accomplishes some conditions requested in previous research to increase the utility of spatial and spatiotemporal analyses [55]. We could identify the neighbourhoods with the highest risk of Covid-19 once socioenvironmental factors and their spatial and temporal distribution are considered, and mapped the variations in risk in each quarter between 2020 and 2022. This provides a risk indicator with higher resolution than previous reports of Covid-19 risk in Cúcuta that can be used to guide control and or prevention strategies in high-risk neighbourhoods or clusters of higher risk. Spatial analyses have been increasingly used to support decision-making in the health sector, from accurate characterisation of areas and population groups [56,57] and better prediction of morbidity outcomes [58] to the targeted use of these methods in control and planning [59]. These applications can be implemented in large urban settings such as Cúcuta where differences in sociodemographic and environmental determinants between small areas such as neighbourhoods justify the use of robust methodologies to support better evidence-based public health interventions.

## 5 Limitations

This study has some limitations, especially related to the data categorised for geographical areas implying a potential risk of ecological bias (i.e., the neighbourhood aggregated data can produce spurious statistical associations). The ecological design of the study means that the risk estimates cannot be interpreted as measures of causality and individual level data would be required to confirm the potential causal links between the sociodemographic factors investigated and the spread of Covid-19 in Cúcuta. In addition, several covariates lack higher spatial resolution because this information is not collected officially by the planning and or health departments and had to be obtained from public online servers. This could have affected the accuracy of the risk estimates for specific geographic areas. To control the risk of ecological bias, apart from adjusting for several sociodemographic factors and the use of a hierarchical Bayesian model, we used the same standardisation method for all rates included in the model, which helps to reduce the effect of the data aggregation in ecological studies [60]. Since the analysis focused on neighbourhoods in Cúcuta, these findings may not be applicable to other cities due to differences in the Covid-19 rate and the predictors distribution in different sociodemographic and geographic characteristics.

While the study uses a Bayesian model to increase the robustness of the estimates, this approach implies some assumptions, especially in the prior selection. To control for potential bias in the prior choice, we conducted a sensitivity analysis to compare four priors previously tested in spatial analyses of Cúcuta spatial areas. Finally, although only the use of individual data can remove completely the risk of ecological bias, this study design is useful for exploratory analyses [61], which is especially enhanced with the incorporation of a spatiotemporal specification to allow mapping the risk for specific geographic areas, a key aim of this analysis.

## 6 Conclusions

Bayesian spatiotemporal analyses identify the number of high-traffic roads, a proxy indicator of mobility and connectivity, as the most important determinant of Covid-19 infection in Cúcuta, Colombia. Other socioenvironmental factors such as the poverty and stringency indexes, PM_10_ and education are overruled by the number of high-traffic roads when the Bayesian model includes a space-time interaction. This study highlights how Bayesian hierarchical regressions can be key to identify significant determinants of Covid-19 in suburban areas of large cities and to support public health decision-making by identifying specific suburban areas with higher risk. Since the study uses data at the neighbourhood level, further research with individual data is warranted to establish causal links of these factors with Covid-19 in addition to validation analyses to allow the generalisation of the results in other Colombian cities. Future studies can expand this research to investigate how these findings can be used in effective health interventions and policies to mitigate the impacts of emerging infectious diseases such as Covid-19.

## Data availability statement

The processed data required to reproduce the above findings are available to download from the supplementary materials included in this paper. These include the data on all covariates and standardised rates of Covid-19. The raw data on the number of Covid-19 cases required to calculate the standardised rates of Covid-19 cannot be shared at this time due to conditions in the ethics approval to use these data.

## CRediT authorship contribution statement

**J. Cortes-Ramirez:** Writing – review & editing, Writing – original draft, Project administration, Methodology, Formal analysis, Conceptualization. **J.D. Wilches-Vega:** Writing – original draft, Formal analysis, Data curation. **B. Caicedo-Velasquez:** Writing – original draft, Validation. **O.M. Paris-Pineda:** Writing – original draft, Validation, Data curation. **P.D. Sly:** Writing – original draft, Validation.

## Declaration of competing interest

The authors declare that they have no known competing financial interests or personal relationships that could have appeared to influence the work reported in this paper.

## Figures and Tables

**Table A1:** Deviance Information Criterion (DIC) of the regression model with each of the 4 priors

	Prior 1	Prior 2	Prior 3	Prior 4
Non-parametric model (model 1)	710177.3	710175.8	710171.5	**710169.7**
Non-parametric model with space time interaction (model 2)	21563.37	21563.4	21563.4	**21562.75**

DIC of the best fit model in bold.

## Appendix

### Preliminary analysis

The following priors were used to identify the effect of the prior on the log of the structured and unstructured spatial precisions in the spatial regression model:

$$Prior\;1:\log\;\tau_{v}∼\log\;Gamma\;\left(1,0.005\right),\log\;\tau_{\upsilon }∼\log\;Gamma\;\left(1,0.005\right)$$

$$Prior\;2:\log\;\tau_{v}∼\log\;Gamma\;\left(0.5,0.001\right),\log\;\tau_{\upsilon }∼\log\;Gamma\;\left(0.5,0.001\right)$$

$$Prior\;3:\log\;\tau_{v}∼\log\;Gamma\;\left(0.1,0.1\right),\log\;\tau_{\upsilon }∼\log\;Gamma\;\left(0.001,0.001\right)$$

$$Prior\;4:\log\;\tau_{v}∼\log\;Gamma\;\left(0.1,0.01\right),\log\;\tau_{\upsilon }∼\log\;Gamma\;\left(0.1,0.01\right)$$
